# Supplementary material for: An outbreak of acute jaundice syndrome (AJS) among the Rohingya refugees in Cox’s Bazar, Bangladesh: Findings from enhanced epidemiological surveillance
Source: PLoS One. 2021 Apr 29;16(4):e0250505. doi: 10.1371/journal.pone.0250505 (PMC8084213; doi:10.1371/journal.pone.0250505)
Supplement: S1 Appendix — (PDF) [file pone.0250505.s001.pdf]

Etiologies of acute Jaundice Syndrome

**Disease**	**Mode of transmission**	**Incubation Period**	**Symptoms**	**Age group at Risk of infection**
Hepatitis A	Feco-oral	2 to 4 weeks [1]	Jaundice (yellow eyes and skin, dark urine), Loss of appetite, Nausea, Fever, Diarrhea, Fatigue, abdominal pain	In endemic countries, infection tends to occur in childhood and most adults are immune.
Acute Hepatitis B/C<br>or acute flare up on chronic Hepatits B/C	Intravenous, Sexual, Vertical	2 weeks to 6 months [2,3]	Initially can be asymptomatic. Those who are acutely symptomatic may exhibit fever, fatigue, decreased appetite, nausea, vomiting, abdominal pain, dark urine, clay/grey-coloured faeces, joint pain and jaundice (yellowing of the skin and the whites of the eyes) (9).	Infants (vertical), adults (sexual transmission), all ages (intravenous). Mostly commonly seen in adults for prolonged duration of infection and delay in diagnosis [4].
Hepatitis E	The virus is transmitted via the fecal-oral route, principally via contaminated water [5]	The incubation period ranges from 2 to 10 weeks, with an average of 5 to 6 weeks	jaundice (yellow colour of the skin and whiteness of the eyes), with dark urine and pale stools, a slightly enlarged tender liver (hepatomegaly) with initially mild fever, reduced appetite (anorexia), nausea and vomiting,	Generally follows the pattern of Hep A. In areas with high disease endemicity, symptomatic infection is most common in young adults aged 15–40 years [5]. Unlike Hep A, there are higher risks to have maternal complications and deaths among pregnant women.
Leptospiros is	Direct Contact with animal excreta. Generally transmitted through environmental exposure.	2 days to 4 weeks [6]	high fever, headache, chills, muscle aches, diarrhea, vomiting, red eyes, abdominal pain, and rash	All. Outbreaks more often seen in adult males when there is environmental exposure (e.g. stagnant water, floods etc). - check references

Reference

1. WHO. Hepatitis A [Internet]. [cited 2019 Dec 15]. Available from: https://www.who.int/news-room/fact-sheets/detail/hepatitis-a

2. WHO. Hepatitis B [Internet]. [cited 2019 Dec 15]. Available from: https://www.who.int/news-room/fact-sheets/detail/hepatitis-b

3. WHO. Hepatitis C [Internet]. [cited 2019 Dec 15]. Available from: https://www.who.int/news-room/fact-sheets/detail/hepatitis-c

4. Reid M, Price JC, Tien PC. Hepatitis C Virus Infection in the Older Patient. Vol. 31, Infectious Disease Clinics of North America. W.B. Saunders; 2017. p. 827–38.

5. WHO. Hepatitis E [Internet]. 2019 [cited 2019 Nov 23]. Available from: https://www.who.int/news-room/fact-sheets/detail/hepatitis-e

6. CDC. Signs and Symptoms | Leptospirosis | CDC [Internet]. [cited 2019 Dec 15]. Available from: https://www.cdc.gov/leptospirosis/symptoms/index.html
