# Supplementary material for: An outbreak of acute jaundice syndrome (AJS) among the Rohingya refugees in Cox’s Bazar, Bangladesh: Findings from enhanced epidemiological surveillance
Source: PLoS One. 2021 Apr 29;16(4):e0250505. doi: 10.1371/journal.pone.0250505 (PMC8084213; doi:10.1371/journal.pone.0250505)
Supplement: S2 Appendix — (PDF) [file pone.0250505.s002.pdf]

Acute Jaundice Syndrome Case Report Form

Location of the health facility*:_______________________________ **Report date** (dd/mm/yy): ____/____/____

Facility number (Leave blank until further notice- health facility number will be provided):____

Case ID* (Today's date (ddmmyy) followed by case number assigned at the facility today; e.g. 1st AJS case at your facility on 15th Feb 18 would be 150218001)

D	D	M	M	Y	Y	**# 0-9**	**# 0-9**	**# 0-9**

Case ID used by the facility if available and different from the case ID above:

I.CASE IDENTIFICATION/ DEMOGRAPHIC DETAILS

Patient name:	Sex*:<br>• Male • Female<br>•Unknown	Date of birth (dd/mm/yy)<br>____/____/_____
Age in years (for children aged less than 12 months, enter 0 as the age in years) :		
Father's /husband's name:	Family card/ Ration card/ RRRC card number:<br>(completion of this field NOT compulsory):	
Family phone number:	Mahji name:	Mahji phone number:
Mosque name:	Imam name:	Work as food handler:<br>• Yes • No • Unknown
Nationality: • FDMN • National • Other		

**For Forcibly Displaced Myanmar National case**		**For National case**
Camp (new camp names, e.g. C7)	Hill (Local description)	Upazilla
Zone (e.g. AA)	Place (Local description)	Division
Block	Latitude (if available)	Union/Ward
House number	Longitude (if available)	

Pregnancy related information

Pregnancy • Yes • No • Unknown If pregnant, which trimester • First • Second • Third
Miscarriage/ stillbirth/ neonatal death during this period of illness • Yes • No • Unknown

II. CLINICAL DETAILS:

Date of jaundice onset (dd/mm/yy)<br>___/_______/______	Date of symptoms onset (dd/mm/yy)<br>___/_______/_______	Date of examination (dd/mm/yy) *<br>___/_______/______
**Symptoms & Signs (history or at admission-tick all that apply)**		
• Fever<br>• Jaundice<br>• Dark Urine<br>• Loss of appetite<br>• Fatigue	• Generalized itch<br>• Joint pain<br>• Nausea<br>• Vomiting<br>• Abdominal pain	• Diarrhoea<br>• Bleeding<br>• Convulsions<br>• Altered Mental state<br>• Other, Specify _____________
Was patient admitted: • Yes • No	Date of admission (dd/mm/yy) ____/_______/______	

III. FAMILY HISTORY:

Number of persons living in the household:<br>_____________	Other people in the household with jaundice in the last 3 months:<br>• Yes • No
If yes, number of people in the household with similar illness (enter number):<br>_____________	Onset of the first case in the household:<br>• <2 weeks ago • 2-<4 weeks ago • 4-<6 weeks ago • 6-<8 weeks ago • 8 weeks or longer

IV.HYGIENE AND SANITATION

Water source most often used (one answer):<br>• Tube well • Communal tap • Tanker truck<br>• Spring water (local word: shora) • Rain water collection<br>• Other, Specify _____________<br><br>Water storage most often used (one answer):<br>• Aluminium/Metal pot • Plastic bucket • Jerry can<br>• Ceramic Jug • Clay pot<br>• Other, Specify _____________<br><br>Use Water purification:	Usually wash hands before taking food:<br>• Always • Sometimes • Never<br><br>Usually wash hands after defecation or changing nappies:<br>• Always • Sometimes • Never<br><br>Use soap to wash hand:<br>• Always • Sometimes • Never<br><br>Usual place of defecation:<br>• Latrine • Open defecation<br>• Other, specify_____________

• Always • Sometimes • Never<br><br>Method of water purification usually used:<br>• Boiling • Filter • Water purification tablets<br>• Other, specify ___________________	Condition of latrine last time used:<br>• Empty • Somewhat filled<br>• Close to overflowing • Overflowing

V. DIAGNOSTIC RESULTS:

**Rapid diagnostic test done*:** • **Yes** • **No**		
If yes, mention the test result and date of the RDT		
RDT	Result	RDT date(dd/mm/yy):
Hepatitis E	• Positive • Negative • Indeterminate • No RDT done	___/_______/_____
Hepatitis B	• Positive • Negative • Indeterminate • No RDT done	___/_______/_____
Hepatitis C	• Positive • Negative • Indeterminate • No RDT done	___/_______/_____
Malaria	• Positive • Negative • Indeterminate • No RDT done	___/_______/_____
If other RDT (Specify):	• Positive • Negative • Indeterminate • No RDT done	___/_______/_____

**Specimen collection done*:** • **Yes** • **No** • **Unknown**		
If yes, type of sample collected: • Blood • Serum	Date of collection (dd/mm/yy): ___/_______/______	
Test	Result	Date of result (dd/mm/yy):
HAV IgM	• Positive • Negative • Indeterminate • No Test done	___/_______/_____
HBsAg	• Positive • Negative • Indeterminate • No Test done	___/_______/_____

HCV IgM	• Positive • Negative • Indeterminate • No Test done	___/_______/_____
HEV IgM	• Positive • Negative • Indeterminate • No Test done	___/_______/_____
Leptospirosis	• Positive • Negative • Indeterminate • No Test done	___/_______/_____
Malaria (Blood Smear)	• Positive • Negative • Indeterminate • No Test done	___/_______/_____
Other test (Specify)	• Positive • Negative • Indeterminate	___/_______/_____

VI. DISCHARGE DETAILS (For admitted patients)

Outcome at discharge:<br>• Discharged • Death • Referred<br>• Left against medical advice • Unknown	Date of Discharge(dd/mm/yy):<br>____ /________/______	Date of Death(dd/mm/yy):<br>____ /________/______
Comments:		
