# Supplementary material for: An outbreak of acute jaundice syndrome (AJS) among the Rohingya refugees in Cox’s Bazar, Bangladesh: Findings from enhanced epidemiological surveillance
Source: PLoS One. 2021 Apr 29;16(4):e0250505. doi: 10.1371/journal.pone.0250505 (PMC8084213; doi:10.1371/journal.pone.0250505)
Supplement: S3 Appendix — (PDF) [file pone.0250505.s003.pdf]

Acute Jaundice Syndrome - Case Definition

Acute Jaundice Syndrome (AJS): Any person with acute onset of jaundice (yellowing of the eyes/skin and/or dark brown urine) with or without fever and without any known precipitating factors.
