# Supplementary material for: An outbreak of acute jaundice syndrome (AJS) among the Rohingya refugees in Cox’s Bazar, Bangladesh: Findings from enhanced epidemiological surveillance
Source: PLoS One. 2021 Apr 29;16(4):e0250505. doi: 10.1371/journal.pone.0250505 (PMC8084213; doi:10.1371/journal.pone.0250505)
Supplement: S4 Appendix — (PDF) [file pone.0250505.s004.pdf]

**Water quality testing results in refugee settlements from 18 September to 14 November 2017, Cox's Bazar, Bangladesh** [1]

Samples were collected from water sources between September and November 2017 showed that 92% of water samples collected from households (n=1638) and 62% of water collected from water sources (n=831) tested positive for fecal contamination with Escherichia coli (E.coli) as per the Bangladesh Standard and WHO guidelines. This indicates high levels of household level contamination after water collection. Of the total positive samples, 43% (855) were very highly contaminated (>100 cfu/100ml) and 28% (571) highly contaminated (>50 and 100 cfu/100ml) [1].

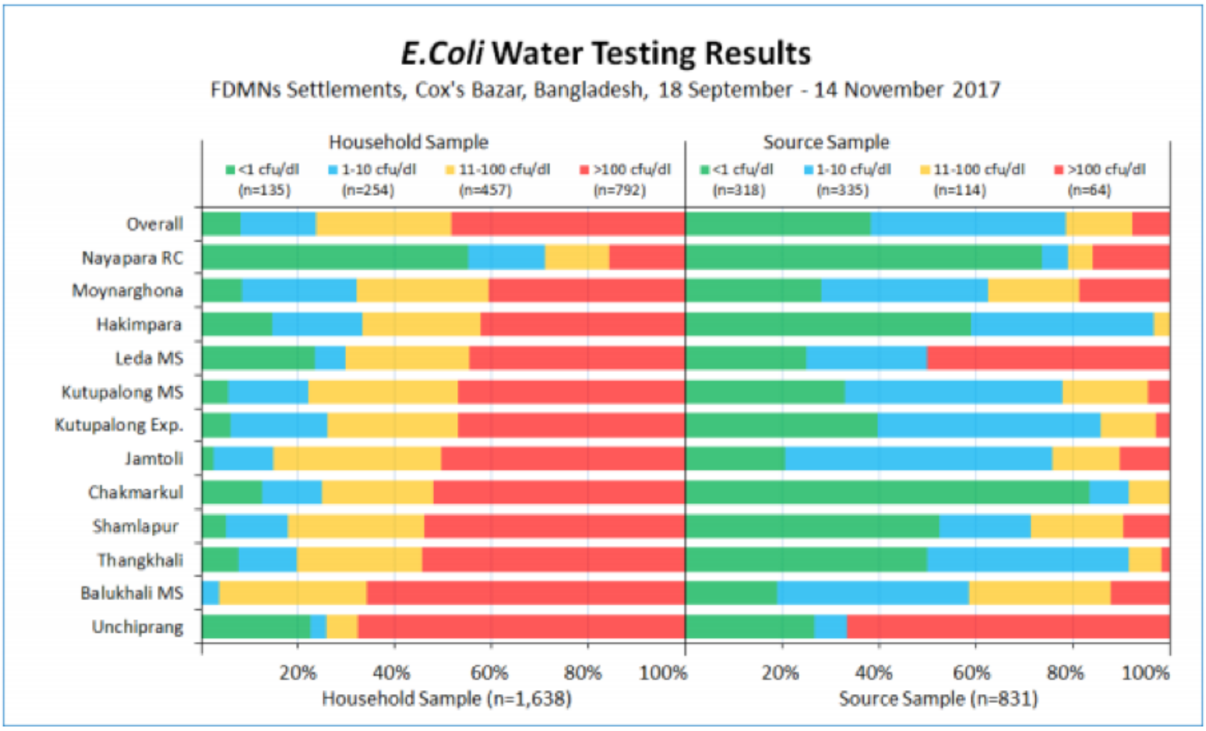


## Reference


1. WHO. Mortality and Morbidity Weekly Bulletin (MMWB).
