# Supplementary material for: An outbreak of acute jaundice syndrome (AJS) among the Rohingya refugees in Cox’s Bazar, Bangladesh: Findings from enhanced epidemiological surveillance
Source: PLoS One. 2021 Apr 29;16(4):e0250505. doi: 10.1371/journal.pone.0250505 (PMC8084213; doi:10.1371/journal.pone.0250505)
Supplement: S6 Appendix — (PDF) [file pone.0250505.s006.pdf]

Reported AJS Household Transmission – Definition

Reported AJS Household Transmission: If any household member presented with similar illness <8 weeks before the onset of jaundice.
