# Supplementary material for: An outbreak of acute jaundice syndrome (AJS) among the Rohingya refugees in Cox’s Bazar, Bangladesh: Findings from enhanced epidemiological surveillance
Source: PLoS One. 2021 Apr 29;16(4):e0250505. doi: 10.1371/journal.pone.0250505 (PMC8084213; doi:10.1371/journal.pone.0250505)
Supplement: Attachment — Submitted filename: Peer review 15.02.21 Dr Tripurari kumar.pdf [file pone.0250505.s009.pdf]

REVIEWER FORM – Comments for the authors

This page WILL be seen by the author(s)

Article ref no.	PONE-D-20-32697
Title	Hepatitis A outbreak among the Rohingya refugees in Cox's Bazar, Bangladesh: findings from enhanced epidemiological surveillance
Author(s)	Md Khadimul Anam Mazhar, MBBS, MPH<br>Flavio Finger<br>Egmond Evers<br>Anna Kuehne<br>Francis Yeasurajan<br>Tahmina Shirin<br>Nurul Ajim<br>Ahammadul Kabir
Referee's name	

When assessing the work, please consider the following points, where applicable:

1. Is the question posed by the authors new and well defined?
2. Are the methods appropriate and well described, and are sufficient details provided to replicate the work?
3. Are the data sound and well controlled?
4. Does the manuscript adhere to the relevant standards for reporting and data deposition?
5. Are the discussion and conclusions well balanced and adequately supported by the data?
6. Do the title and abstract accurately convey what has been found?
7. Is the writing acceptable?

Please make your report as constructive and detailed as possible in your comments so that authors have the opportunity to overcome any serious deficiencies that you find and please also divide your comments into the following categories:

- Major Compulsory Revisions (which the author must respond to before a decision on publication can be reached)
- Minor Essential Revisions (such as missing labels on figures, or the wrong use of a term, which the author can be trusted to correct)
- Discretionary Revisions (which are recommendations for improvement but which the author can choose to ignore)

Where possible please supply references to substantiate your comments. When referring to the manuscript please provide specific page and paragraph citations where appropriate. Continue on another sheet if needed.

General comments **Now the article looks more structured and need few editing and formatting.**

Minor compulsory revisions:

All the comments on the paper need to be addressed as follows:

A. Result section:

1) In Table 3, Please mention which age group in first row???

	N (%)	Odds Ratio (95% CI)	P-value
Age (in years)	275 (100%)	0.86 (0.83 to 0.89)	< 0.001,

This table shows two sub categories, age & gender. please make age data together and then put gender data

In Table 4, pl put the data as per no. of cases presented with symptoms like fever most common presented

(n=183) followed by Nausea (n=155) and abdominal pain (n=113) and so on..

Map Section: **1.** AR should be calculated age-wise, sex wise and location wise.

2**.** Map of camps are shown as no. of cases with different gradients, it should be depicted as per Attack rate (No of AJS cases in camp wise/ total population of given camp %),

3. We can see there were 34 camps and 18 health facilities, for better interpretation, map can be merged as per catering population by each health facility and calculate AR accordingly.

Discussion Section:

1. As per topic heading is concerned, "**Hepatitis A outbreak among the Rohingya refugees in Cox's Bazar, Bangladesh:findings from enhanced epidemiological surveillance";** it's better to keep **"Jaundice outbreak among the Rohingya refugees in Cox's Bazar, Bangladesh: findings from enhanced epidemiological surveillance"-** there were cases **multifactorial etiologies found after investigation, so in my opinion Jaundice outbreak covers all details.**
2. Regarding Hep B cases found during surveillance, warrants immunization Hep- B drive in all age group healthy individuals in 34 camps as well as zero doses to new borne ASAP to protect from spread and close monitoring to cases.
3. In last, I feel for better representation of data, descriptive data should be kept separate and analytical data should be separate.

Please send word documents more comments on track change mode.

The paper could be improved by few editing and formatting.
