# Supplementary material for: An outbreak of acute jaundice syndrome (AJS) among the Rohingya refugees in Cox’s Bazar, Bangladesh: Findings from enhanced epidemiological surveillance
Source: PLoS One. 2021 Apr 29;16(4):e0250505. doi: 10.1371/journal.pone.0250505 (PMC8084213; doi:10.1371/journal.pone.0250505)
Supplement: Attachment — Submitted filename: Peer review form peer review Dr Tripurari kumar.docx [file pone.0250505.s007.docx]

| REVIEWER FORM – Comments for the authors  This page WILL be seen by the author(s) | |
| --- | --- |
| Article ref no. | PONE-D-20-32697 |
| Title | Hepatitis A outbreak among the Rohingya refugees in Cox’s Bazar, Bangladesh: findings from enhanced epidemiological surveillance |
| Author(s) | Md Khadimul Anam Mazhar, MBBS, MPH  Flavio Finger  Egmond Evers  Anna Kuehne  Francis Yeasurajan  Tahmina Shirin  Nurul Ajim  Ahammadul Kabir |
| Referee’s name |  |
| When assessing the work, please consider the following points, where applicable:   1. Is the question posed by the authors new and well defined? 2. Are the methods appropriate and well described, and are sufficient details provided to replicate the work? 3. Are the data sound and well controlled? 4. Does the manuscript adhere to the relevant standards for reporting and data deposition? 5. Are the discussion and conclusions well balanced and adequately supported by the data? 6. Do the title and abstract accurately convey what has been found? 7. Is the writing acceptable?   Please make your report as constructive and detailed as possible in your comments so that authors have the opportunity to overcome any serious deficiencies that you find and please also divide your comments into the following categories:  Major Compulsory Revisions (which the author must respond to before a decision on publication can be reached)  Minor Essential Revisions (such as missing labels on figures, or the wrong use of a term, which the author can be trusted to correct)   - Discretionary Revisions (which are recommendations for improvement but which the author can choose to ignore)   Where possible please supply references to substantiate your comments. When referring to the manuscript please provide specific page and paragraph citations where appropriate. Continue on another sheet if needed. | |
| General comments  This is a very important topic of a significant public health problem in the huge migrations / refugee crises in developing world like in Bangladesh. This looks one-month (28^th^ Feb to 24 March 2018) enhanced surveillance data analysis.  This AJS exemplifies what is continuously happening in most parts of the world where forced migrations/ border conflicts or refugees’ camps due to lack of shelter, food, safe drinking water and better sanitation. | |
| **Major compulsory revisions**  **All the comments on the paper need to be addressed as follows:**  1. As the refugees migration took place in Summer of 2017 means May, June as starting point and as per epi curve the data of AJS cases showed approx.. more than 3500 cases and 6 deaths but 6 deaths were not shown and not mentioned in this articles, please include  2. Location map is very important to look for clustering of cases in camps. As per Epi-curve, there is gradually increase of no. of health units under surveillance (EWARS) approx. 160 units but data had been shown to only 18 health units for 4 weeks, most probably because of more cases in Feb to March 2018 in those units, but we missed which parts of camp are more affected and we can explain better if we put map of location and camps in COX’s bazar.  3. We cannot comment odd ratio without table and percentage: Naked odds ratios give no idea about the extent of this risk factor. It’s always better to include the percent of cases and percent of controls exposed. One could get an OR of 5 with only 20% exposed.  **Please send word documents more comments on track change mode.** | |
| **Minor essential revisions**  **The paper could be improved by thorough editing and formatting.** | |
