# Supplementary material for: An outbreak of acute jaundice syndrome (AJS) among the Rohingya refugees in Cox’s Bazar, Bangladesh: Findings from enhanced epidemiological surveillance
Source: PLoS One. 2021 Apr 29;16(4):e0250505. doi: 10.1371/journal.pone.0250505 (PMC8084213; doi:10.1371/journal.pone.0250505)
Supplement: Attachment — Submitted filename: Response to Reviewers (updated).docx [file pone.0250505.s008.docx]

# Response to Reviewers:

## Journal Requirements:

A. **When submitting your revision, we need you to address these additional requirements.**

**1. Please ensure that your manuscript meets PLOS ONE's style requirements, including those for file naming. The PLOS ONE style templates can be found at**

[**https://journals.plos.org/plosone/s/file?id=wjVg/PLOSOne_formatting_sample_main_body.pdf**](https://journals.plos.org/plosone/s/file?id=wjVg/PLOSOne_formatting_sample_main_body.pdf) **and**

[**https://journals.plos.org/plosone/s/file?id=ba62/PLOSOne_formatting_sample_title_authors_affiliations.pdf**](https://journals.plos.org/plosone/s/file?id=ba62/PLOSOne_formatting_sample_title_authors_affiliations.pdf)

We thank the reviewers for drawing attention to meet PLOS One’s style requirements. We have made necessary amendments in the manuscript and file naming during our re-submission.

**2. Thank you for including your ethics statement provided within your manuscript:**

**"The data reported were collected as public health outbreak response activities. For this 180publication, no additional primary data collection took place. However, theanalysis was done with the support of and in consultation withthe Civil Surgeon, Cox’s Bazar, Bangladesh. All individual case data was anonymized."**

**a. Please amend your current ethics statement to include the full name of the ethics committee/institutional review board(s) that approved your specific study.**

**b. Once you have amended this/these statement(s) in the Methods section of the manuscript, please add the same text to the “Ethics Statement” field of the submission form (via “Edit Submission”).**

**For additional information about PLOS ONE ethical requirements for human subjects research, please refer to** [**http://journals.plos.org/plosone/s/submission-guidelines#loc-human-subjects-research**](http://journals.plos.org/plosone/s/submission-guidelines#loc-human-subjects-research)**.**

We thank the reviewers for drawing attention to ethics statement. The data reported were collected as part of routine public health outbreak response activities. For this publication, no additional primary data collection took place other than the data required for outbreak response and surveillance. The formulation of this work was presented, reviewed and approved by the review committee for “Operational research during Rohingya Refugee crisis” is led by the Ministry of Health & Family Welfare (MoHFW), Bangladesh. Written approval to publish this manuscript in a journal was obtained from the Ministry of Health & Family Welfare (MoHFW), Bangladesh responsible for the oversight the response of the refugee crisis in Cox’s Bazar, Bangladesh. We have updated the ethics statement as follows as follows (lines 190 to 197):

“*The data reported were collected as part of routine public health outbreak response activities. For this publication, no additional primary data collection took place other than the data required for outbreak response and surveillance. Therefore, no additional ethical approval was sought at the time of data collection. The formulation of this work was presented and discussed with the Civil Surgeon Cox’s Bazar from the Ministry of Health & Family Welfare (MoHFW) responsible for overseeing the Rohingya refugee crisis. All individual case data was anonymized.”*

**3. Please provide additional details regarding participant consent.**

**In the ethics statement in the Methods and online submission information, please ensure that you have specified (i) whether consent was informed and (ii) what type you obtained (for instance, written or verbal, and if verbal, how it was documented and witnessed). If your study included minors, state whether you obtained consent from parents or guardians. If the need for consent was waived by the ethics committee, please include this information.**

**If you are reporting a retrospective study of medical records or archived samples, please ensure that you have discussed whether all data were fully anonymized before you accessed them and/or whether the IRB or ethics committee waived the requirement for informed consent. If patients provided informed written consent to have data from their medical records used in research, please include this information.**

We thank the reviewers for drawing attention regarding participant consent. We have updated the text regarding participants consent (lines 155 to 158):

*“Participants were provided with information about the reason for the blood tests, and how their information would be handled and stored. Verbal consent was obtained by the medical team in charge at each of the 18 participating facilities. Participants who refused to provide consent, were excluded from sample collection.”*

**4. We note that you have indicated that data from this study are available upon request. PLOS only allows data to be available upon request if there are legal or ethical restrictions on sharing data publicly. For information on unacceptable data access restrictions, please see** [**http://journals.plos.org/plosone/s/data-availability#loc-unacceptable-data-access-restrictions**](http://journals.plos.org/plosone/s/data-availability#loc-unacceptable-data-access-restrictions)**.**

**In your revised cover letter, please address the following prompts:**

**a) If there are ethical or legal restrictions on sharing a de-identified data set, please explain them in detail (e.g., data contain potentially identifying or sensitive patient information) and who has imposed them (e.g., an ethics committee). Please also provide contact information for a data access committee, ethics committee, or other institutional body to which data requests may be sent.**

**b) If there are no restrictions, please upload the minimal anonymized data set necessary to replicate your study findings as either Supporting Information files or to a stable, public repository and provide us with the relevant URLs, DOIs, or accession numbers. Please see** [**http://www.bmj.com/content/340/bmj.c181.long**](http://www.bmj.com/content/340/bmj.c181.long) **for guidelines on how to de-identify and prepare clinical data for publication. For a list of acceptable repositories, please see** [**http://journals.plos.org/plosone/s/data-availability#loc-recommended-repositories**](http://journals.plos.org/plosone/s/data-availability#loc-recommended-repositories)**.**

**We will update your Data Availability statement on your behalf to reflect the information you provide.**

We thank the reviewers for drawing attention to data availability statement. The data used to develop the manuscript related to the Rohingya refugee population living in Cox’s Bazar and are owned by Ministry of Health & Family Welfare (MoHFW). Due to the political sensitivity of the subject matter, there is restriction on sharing information on Rohingya refugee population. A written approval was obtained from the MoHFW to use this anonymised data set only for the purpose of sharing the results of this outbreak investigation and in the context of this manuscript. Any further request to access the data has to be approved by the MoHFW responsible for overseeing the Rohingya refugee response. The contact details are given below –

- Civil Surgeon Office, Ministry of Health & Family Welfare,
- Kolatoli Road, Cox’s Bazar, Bangladesh
- Email: [coxsbazar@cs.dghs.gov.bd](mailto:coxsbazar@cs.dghs.gov.bd)

**5. Thank you for stating the following financial disclosure:**

**'The funders had no role in study design, data collection and analysis, decision to publish, or preparation of the manuscript'**

**At this time, please address the following queries:**

a. **Please clarify the sources of funding (financial or material support) for your study. List the grants or organizations that supported your study, including funding received from your institution.**
b. **State what role the funders took in the study. If the funders had no role in your study, please state: “The funders had no role in study design, data collection and analysis, decision to publish, or preparation of the manuscript.”**
c. **If any authors received a salary from any of your funders, please state which authors and which funders.**
d. **If you did not receive any funding for this study, please state: “The authors received no specific funding for this work.”**

**Please include your amended statements within your cover letter; we will change the online submission form on your behalf.**

We thank the reviewers for drawing attention to financial disclosure. We have made following changes in our manuscript (lines: 366 to 373) –

*“The authors received no specific funding for this work. The UK Public Health Rapid Support Team is funded by UK Aid from the Department of Health and Social Care and is jointly run by Public Health England and the London School of Hygiene & Tropical Medicine. The views expressed in this publication are those of the authors and not necessarily those of the National Health Service, the National Institute for Health Research or the Department of Health and Social Care. The funders had no role in study design, data collection and analysis, decision to publish, or preparation of the manuscript.”*

**6. Please include a separate caption for each figure in your manuscript.**

We thank the reviewers for drawing attention to this. We have made changes in to the manuscript accordingly.

**7. Please ensure that you refer to Figure 2 in your text as, if accepted, production will need this reference to link the reader to the figure.**

We thank the reviewers for drawing attention to this. We have made changes in to the manuscript accordingly (lines: 104).

**8. We note that Supporting Information Appendix 3 in your submission contains map images which may be copyrighted.**

**All PLOS content is published under the Creative Commons Attribution License (CC BY 4.0), which means that the manuscript, images, and Supporting Information files will be freely available online, and any third party is permitted to access, download, copy, distribute, and use these materials in any way, even commercially, with proper attribution. For these reasons, we cannot publish previously copyrighted maps or satellite images created using proprietary data, such as Google software (Google Maps, Street View, and Earth). For more information, see our copyright guidelines:** [**http://journals.plos.org/plosone/s/licenses-and-copyright**](http://journals.plos.org/plosone/s/licenses-and-copyright)**.**

**We require you to either (a) present written permission from the copyright holder to publish these figure specifically under the CC BY 4.0 license, or (b) remove the figure from your submission:**

**a. You may seek permission from the original copyright holder of Supporting Information Appendix 3 to publish the content specifically under the CC BY 4.0 license.**

**We recommend that you contact the original copyright holder with the Content Permission Form (http://journals.plos.org/plosone/s/file?id=7c09/content-permission-form.pdf) and the following text:**

**“I request permission for the open-access journal PLOS ONE to publish XXX under the Creative Commons Attribution License (CCAL) CC BY 4.0 (http://creativecommons.org/licenses/by/4.0/). Please be aware that this license allows unrestricted use and distribution, even commercially, by third parties. Please reply and provide explicit written permission to publish XXX under a CC BY license and complete the attached form.”**

**Please upload the completed Content Permission Form or other proof of granted permissions as an "Other" file with your submission.**

**In the figure caption of the copyrighted figure, please include the following text: “Reprinted from [ref] under a CC BY license, with permission from [name of publisher], original copyright [original copyright year].”**

**b. If you are unable to obtain permission from the original copyright holder to publish this figure under the CC BY 4.0 license or if the copyright holder’s requirements are incompatible with the CC BY 4.0 license, please either i) remove the figure or ii) supply a replacement figure that complies with the CC BY 4.0 license. Please check copyright information on all replacement figures and update the figure caption with source information. If applicable, please specify in the figure caption text when a figure is similar but not identical to the original image and is therefore for illustrative purposes only.**

**The following resources for replacing copyrighted map figures may be helpful:**

**USGS National Map Viewer (public domain): http://viewer.nationalmap.gov/viewer/**

**The Gateway to Astronaut Photography of Earth (public domain): http://eol.jsc.nasa.gov/sseop/clickmap/**

**Maps at the CIA (public domain): https://www.cia.gov/library/publications/the-world-factbook/index.html and https://www.cia.gov/library/publications/cia-maps-publications/index.html**

**NASA Earth Observatory (public domain): http://earthobservatory.nasa.gov/**

**Landsat:** [**http://landsat.visibleearth.nasa.gov/**](http://landsat.visibleearth.nasa.gov/)

**USGS EROS (Earth Resources Observatory and Science (EROS) Center) (public domain): http://eros.usgs.gov/#**

**Natural Earth (public domain):** [**http://www.naturalearthdata.com/**](http://www.naturalearthdata.com/)

We thank the reviewers for drawing attention to the copyright issues regarding use of maps in the manuscript. The corresponding author is solely the copyright holder of the maps used in the manuscript. Additional copyright supporting document is also provided with the revised submission.

**9. Please include captions for your Supporting Information files at the end of your manuscript, and update any in-text citations to match accordingly. Please see our Supporting Information guidelines for more information:** [**http://journals.plos.org/plosone/s/supporting-information**](http://journals.plos.org/plosone/s/supporting-information)

We thank the reviewers for drawing attention to this. We have made changes in to the manuscript. We added the segment of supporting information in the end of the manuscript (lines: 435 to 443)

B. **Reviewers' comments:**

**It is an article that describes an outbreak of viral hepatitis A associated with poor sanitary conditions in the refugee population. The presence of other etiologies, such as HBV, may be due to the fact that they already had a previous infection, considering that they are endemic areas; however, a comment could be added on the horizontal transmission of HBV in conglomerates of people. A map of the refugee location could be included.
It is important to improve bibliographic references in general. The data in the tables and graphs are repeated and could be described in the text. If it is published it could be like a short communication.**

We thank the reviewers for their valuable comments. We have added a comment on the horizontal transmission of HBV in the manuscript (line 330 to 333):

*“Presence of high number of Hepatitis B in the younger age-groups indicated the need for improving immunization coverage of Hepatitis B among women of reproductive age (WRA) and among all newborns in the camps and the host community by strengthening routine immunization (4). This might in turn reduce the perinatal transmission of hepatitis viruses (18).”*

We thank the reviewers for their suggestion to add a map of refugee location. We added an additional map showing the location of refugee population density in the camps titled – “Fig 1. Camp-wise distribution of the Rohingya refugee population located in Cox’s Bazar, Bangladesh” .

*“ Fig 1. Camp-wise distribution of the Rohingya refugee population located in Cox’s Bazar, Bangladesh*

”

We have also made changes according to the reviewer’s suggestion on bibliographic reference in general.

**Major compulsory revisions**

**All the comments on the paper need to be addressed as follows:**

##### **1.     As the refugees migration took place in Summer of 2017 means May, June as starting point and as per epi curve the data of AJS cases showed approx. more than 3500 cases and 6 deaths but 6 deaths were not shown and not mentioned in this article, please include**

 
 We thank the reviewers for their suggestion and added deaths in the epi curve titled – “Fig 2. Epidemiology curve of reported AJS cases against number of facilities reporting (monthly average) in EWAR system; and timing of changes to the surveillance system from daily to weekly”:

“*Fig 2. Epidemiology curve of reported AJS cases against number of facilities reporting (monthly average) in EWAR system; and timing of changes to the surveillance system from daily to weekly*

”

##### **2.     Location map is very important to look for clustering of cases in camps. As per Epi-curve, there is gradually increase of no. of health units under surveillance (EWARS) approx. 160 units but data had been shown to only 18 health units for 4 weeks, most probably because of more cases in Feb to March 2018 in those units, but we missed which parts of camp are more affected and we can explain better if we put map of location and camps in COX’s bazar.**

We thank the reviewers for their suggestion, however due to resource constraints and lack of laboratory/diagnostic unit, it wasn’t possible to include all health units reporting to our surveillance network. We had to select only a handful of primary care units with diagnostic/laboratory unit (for collecting and processing blood samples).

##### **3.     We cannot comment odd ratio without table and percentage: Naked odds ratios give no idea about the extent of this risk factor.  It’s always better to include the percent of cases and percent of controls exposed.  One could get an OR of 5 with only 20% exposed.**

We thank the reviewers for their comments and we made necessary changes in to the manuscript.

**Please send word documents more comments on track change mode.**

**Minor essential revisions**

**The paper could be improved by thorough editing and formatting.**

We thank the reviewers for their feedback. We have tried to improve our manuscript in our re-revised version.
