# Supplementary material for: An outbreak of acute jaundice syndrome (AJS) among the Rohingya refugees in Cox’s Bazar, Bangladesh: Findings from enhanced epidemiological surveillance
Source: PLoS One. 2021 Apr 29;16(4):e0250505. doi: 10.1371/journal.pone.0250505 (PMC8084213; doi:10.1371/journal.pone.0250505)
Supplement: Attachment — Submitted filename: Response to Reviewers.docx [file pone.0250505.s010.docx]

# Response to Reviewers:

==============================
1. ACADEMIC EDITOR:

- sorry I missed it in the initial submission, but can you explain the fatalities/death demonstrated in figure 1, and what is the purpuse of showing death, while you do not mention death otherwise in manuscript.
- We thank the reviewer for his feedback on this. In our initial manuscript, we did not include the deaths in the epidemiological curve as deaths were reported outside our enhanced surveillance period. However, as suggested by the reviewer as added the deaths in the graph. The authors agreed to remove the deaths as suggested by the academic reviewer as it is difficult to interpret (lines 100 to 103):

*Fig 1. Epidemiology curve of reported AJS cases against number of facilities reporting (monthly average) in EWAR system; and timing of changes to the surveillance system from daily to weekly*

- Another good point by one reviewed, also missed in first go-around, the title may best include jaundice as starting point, and that HAV was found as dominant culprit.
- We thank reviewer for the valuable suggestion. We have changed the title of our manuscript as suggested (lines 1 to 2) –

*“An outbreak of acute jaundice syndrome (AJS) among the Rohingya refugees in Cox’s Bazar, Bangladesh: findings from enhanced epidemiological surveillance.”*

- Also, did you had an HCV IgM test available? I am not aware that such test exist.
- We thank the reviewer for pointing on the issue regarding HCV testing. During the epidemiological investigation, we used the “Biokit bioelisa HCV 4.0” ELISA kit, which uses anti HCV autoantibodies for detecting antigen against HCV from blood samples. The list of tests used with the name of the manufacturer is given below -

| *No* | *Disease/Pathogen* | *Diagnostic test to be performed* |
| --- | --- | --- |
| *1* | *Hepatitis A Virus* | *HAV ELISA for IgM (CTK Biotech)* |
| *2* | *Hepatitis B Virus* | *Hepatitis B surface antigen (RPC Diagnostics)* |
| *3* | *Hepatitis C Virus* | *Anti-HCV antibodies (Biokit bioelisa HCV 4.0)* |
| *4* | *Hepatitis E Virus* | *Anti-HEV ELISA for IgM (CTK Biotech & Euroimmun)* |
| *5* | *Leptospirosis* | *Leptospira lateral flow for IgM (LifeAssay)* |

- We made the following changes in the manuscript (lines 168 to 169) –

| ***No*** | ***Disease/Pathogen*** | ***Diagnostic test to be performed*** |
| --- | --- | --- |
| *1* | *Hepatitis A Virus* | *HAV ELISA for IgM* |
| *2* | *Hepatitis B Virus* | *Hepatitis B surface antigen* |
| *3* | *Hepatitis C Virus* | *Anti-HCV antibodies* |
| *4* | *Hepatitis E Virus* | *Anti-HEV ELISA for IgM* |
| *5* | *Leptospirosis* | *Leptospira lateral flow for IgM* |

- I believe no one was HBV anti-HBc IgM positive?
- We only used Hepatitis B surface antigen (HBsAg) testing for the detection of HBV cases.

==============================

**Minor compulsory revisions:**

**All the comments on the paper need to be addressed as follows:**

A. **Result section:**

**1) In Table 3, Please mention which age group in first row???**

**N (%)	Odds Ratio (95% CI)	P-value**

**Age (in years)	275 (100%)	0.86 (0.83 to 0.89)	< 0.001**

**This table shows two sub categories, age & gender. please make age data together and then put gender data**

- We thank reviewer for his valuable suggestion. We used age (in years) as numerical variable initially and age-group as categorical variable. However, we have made following changes in the manuscript to accommodate the suggested changes and merged 2 tables together to have same format (lines 256 to 259) –

***“Table 4. Association of hepatitis A seropositivity with age-group, gender, presenting symptoms and drinking water sources***

|  | ***OR (95% CI)*** | ***P-value*** |
| --- | --- | --- |
| ***Age-group*** | | |
| *0-4 years (n=67)* | *1.0** | *-* |
| *5-9 years (n=59)* | *1.15 (0.40 to 3.13)* | *0.79* |
| *10-17 years (n=60)* | *0.18 (0.07 to 0.42)* | *< 0.001* |
| *18+ years (n=89)* | *0.02 (0.01 to 0.06)* | *< 0.001* |
| ***Gender*** | | |
| *Female (n=119)* | *1.0** | *-* |
| *Male (n=156)* | *1.32 (0.82 to 2.14)* | *0.256* |
| ***Presenting symptoms****^#^* | | |
| *Fever (n=183)* | *2.25 (1.35 to 3.76)* | *0.002* |
| *Nausea (n=155)* | *1.19 (0.76 to 1.93)* | *0.479* |
| *Abdominal pain (n=113)* | *0.81 (0.50 to 1.31)* | *0.385* |
| *Vomiting (n=70)* | *1.85 (1.05 to 3.27)* | *0.034* |
| *Fatigue (n=57)* | *0.83 (0.46 to 1.50)* | *0.542* |
| *Itching (n=38)* | *1.23 (0.61 to 2.47)* | *0.563* |
| *Joint pain (n=37)* | *0.42 (0.21 to 0.86)* | *0.018* |
| *Loss of Appetite (n=27)* | *3.83 (1.40 to 10.45)* | *0.009* |
| *Dark Urine (n=26)* | *3.63 (1.32 to 9.94)* | *0.012* |
| *Bleeding (n=7)* | *1.04 (0.23 to 4.74)* | *0.960* |
| *Convulsion (n=6)* | *0.38 (0.07 to 2.12)* | *0.271* |
| ***Drinking water sources^^^*** | | |
| *Tube-well (n=170)* | *1.27 (0.78 to 2.06)* | *0.342* |
| *Tap water (n=45)* | *1.22 (0.63 to 2.33)* | *0.555* |
| *Unknown sources (n=58)* | *0.56 (0.32 to 1.01)* | *0.055* |

**Baseline category, ^#^Diarrhoea (n=1) and other (n=2) symptoms were omitted from the summary table, ^^^ water truck (n=2) was omitted from the summary table”*

**In Table 4, pl put the data as per no. of cases presented with symptoms like fever most common presented (n=183) followed by Nausea (n=155) and abdominal pain (n=113) and so on..**

- We thank reviewer for this suggestion on data presentation. We have made the changes in the manuscript as suggested lines (256 to 259).

**Map Section:**

1. **AR should be calculated age-wise, sex wise and location wise.**

- We thank reviewer for the valuable suggestion. We have calculated the attack rates and incorporated in the manuscript (lines 205 to 215) –

*“Camp-wise weekly attack rate (AR) per 10,000 population was calculated for the reported 275 AJS cases detected by the enhanced epidemiological surveillance strategy between 28 February and 26 March 2018. The weekly mean attack rate over the enhanced surveillance period (28 February to 26 March 2018) was 0.60 (95% CI: 0.28 to 0.92) per 10,000 population, across all the camps for the reported AJS cases. However, the AR varies among age-groups and was higher in younger age-groups (see Table 3). AR (per 10,000 population) for different age-groups is graphically presented in Appendix 5.*

***Table 3: Attack rate of reported AJS cases by age-group^*^ and gender among all 34 camps between 28 February and 26 March 2018 in Cox’s Bazar, Bangladesh***

|  | ***Weekly AR/10,000 population (95% CI)*** |
| --- | --- |
| *Overall* | *0.60 (0.28 to 0.92)* |
| ***By age-group*** | |
| *0-4 years* | *0.80 (0.34 to 1.26)* |
| *5-11 years* | *0.73 (0.36 to 1.10)* |
| *12-17 years* | *0.71 (0.31 to 1.11)* |
| *18+ years* | *0.43 (0.15 to 0.92)* |
| ***By gender*** | |
| *Female* | *0.49 (0.21 to 0.77)* |
| *Male* | *0.71 (0.33 to 1.09)* |

** Population data from UNHCR Bangladesh Operational Update, 1 - 15 August 2018, was used to calculate the AJS attack rate (14).”*

*“*

2. **Map of camps are shown as no. of cases with different gradients, it should be depicted as per Attack rate (No of AJS cases in camp wise/ total population of given camp %),**

- We thank reviewer for the suggestion. We have created the following maps depicting attack rates in different camps (lines 100 to 103 and 454 to 457) –

*Fig 1. Epidemiology curve of reported AJS cases against number of facilities reporting (monthly average) in EWAR system; and timing of changes to the surveillance system from daily to weekly*

*Appendix 5. Map of AJS attack rate (per 10,000 population) by age-groups reported during enhanced epidemiological surveillance strategy (between 28 February and 26 March 2018) in the Rohingya refugee camps in Cox’s Bazar, Bangladesh “*

3. **We can see there were 34 camps and 18 health facilities, for better interpretation, map can be merged as per catering population by each health facility and calculate AR accordingly.**

- We thank the reviewer for his thoughtful suggestion. However, we do not have data on clinics’ catchment area as it is very hard to determine catchment area by facility in the camps. We used camp-wise population data to calculate attack rate per only.

**Discussion Section:**

1. **As per topic heading is concerned, “Hepatitis A outbreak among the Rohingya refugees in Cox’s Bazar, Bangladesh:findings from enhanced epidemiological surveillance”; it’s better to keep “Jaundice outbreak among the Rohingya refugees in Cox’s Bazar, Bangladesh: findings from enhanced epidemiological surveillance”- there were cases multifactorial etiologies found after investigation, so in my opinion Jaundice outbreak covers all details.**

- We thank reviewer for his suggestion. We have amended our title as follows (lines 1 to 2) –

*“An outbreak of acute jaundice syndrome (AJS) among the Rohingya refugees in Cox’s Bazar, Bangladesh: findings from enhanced epidemiological surveillance.”*

2. **Regarding Hep B cases found during surveillance, warrants immunization Hep- B drive in all age group healthy individuals in 34 camps as well as zero doses to new borne ASAP to protect from spread and close monitoring to cases.**

- We thank reviewer for this suggestion on the talking point for hepatitis B cases in the discussion section. We made the following changes as suggested by the reviewer (lines 321 to 331)–

*“While Hepatitis B and C were not drivers of the AJS epidemic, the detection of significant number of infections requires attention and likely reflects high prevalence of chronic infections. l. One small study has shown significant prevalence of HBV and HCV among the Rohingya population in Bangladesh (15) and warrants further serological investigation in particular given limited capacity for clinical management within the district. Hepatitis B vaccination is part of the routine EPI programmes in the camps, and our results highlight the need to ensure adequate coverage, as well as a zero dose to new-borns to protect from spread of the infection, with close monitoring of identified cases. There is also need to maintain routine immunization programmes, to improve vaccine coverage of Hepatitis B among women of reproductive age (WRA) and under-five children both in the camps and the surrounding host community (4). This might, in turn, reduce the perinatal transmission of hepatitis viruses (19).”*

3. **In last, I feel for better representation of data, descriptive data should be kept separate and analytical data should be separate.**

- We thank reviewer for this suggestion. We made necessary changes in the manuscript.

Please send word documents more comments on track change mode.

The paper could be improved by few editing and formatting.

- We thank reviewer for this valuable suggestion. We also tried to improve the manuscript in our revised version. We have made some changes in the discussion segment to make it more focused given all the changes.
